# Do Predictors of Health Facility Delivery Among Reproductive-Age Women Differ by Health Insurance Enrollment? A Multi-Level Analysis of Nigeria's Data

**DOI:** 10.3389/fpubh.2022.797272

**Published:** 2022-04-14

**Authors:** Xiaomei Zhang, Muhammad Khalid Anser, Rolle Remi Ahuru, Zizai Zhang, Michael Yao-Ping Peng, Romanus Osabohien, Mumal Mirza

**Affiliations:** ^1^School of Humanities, Arts and Education, Shandong Xiehe University, Jinan, China; ^2^School of Public Administration, Xi'an University of Architecture and Technology, Xi'an, China; ^3^Department of Economics, Faculty of Social Sciences, University of Benin, Benin City, Nigeria; ^4^Hangzhou Preschool Teachers College, Zhejiang Normal University, Hangzhou, China; ^5^School of Economics and Management, Foshan University, Foshan, China; ^6^Department of Economics and Development Studies, Covenant University, Ota, Nigeria; ^7^Centre for Economics and Development Studies, Covenant University, Ota, Nigeria; ^8^Honorary Research Fellow, ILMA University, Karachi, Pakistan; ^9^Department of Media Science, ILMA University, Karachi, Pakistan

**Keywords:** predictors, health facility delivery, women, health insurance, Nigeria

## Abstract

This study aims to compare determinants of health facility delivery for women under a health insurance scheme and those not under a health insurance scheme. Secondary data drawn from the National Demographic and Health Survey was used for the analysis. The characteristics of the women were presented with simple proportions. Binary multilevel logistic regression was used to examine the determinants of health facilities for women who enrolled in health insurance and those who did not. All statistical analyses were set at 5% level of significant level (*p* = 0.24). The result showed that 2.1% of the women were under a health insurance scheme. Disparity exists in health insurance ownership as a higher proportion of those enrolled in health insurance were those with higher education attainment, in urban parts of the country, and those situated on higher wealth quintiles. There is a significant difference between those with and those without health insurance. It implies that a higher proportion of women who enrolled in health insurance delivered in health facility delivery compared to those who do not. The unique determinants of health facility delivery for women under health insurance were parity and birth order, while unique determinants of health facility delivery for women not enrolled in health schemes were employment status, marriage type, and geopolitical zones. Uniform predictors of health facility delivery for both groups of women were maternal education, household wealth quintiles, autonomy on healthcare, number of antenatal contacts, residential status, community-level poverty, community-level media use, and community-level literacy. Intervention programs designed to improve health facility delivery should expand educational opportunities for women, improve household socioeconomic conditions, target rural women, and encourage women to undertake a minimum of four antenatal contacts.

## Introduction

According to estimates, over 40% of all pregnancies may experience some form of complication. Hence, women are advised to deliver their babies in health institutions, where all possible complications can be resolved (1–3). Recent evidence shows that almost all deliveries in developed countries are supervised in health institutions, and this in part accounts for an extremely low rate of maternal death among developed countries (4). On the contrary, there is predominance of non-institutional delivery among women in developing countries (5–8). Increasing the proportion of deliveries conducted in a health facility is an important health strategy to reduce high maternal mortality rates in developing countries, particularly in Africa (9, 10).

An important health and social policy implemented among developing countries to increase the coverage of health facility delivery is health insurance schemes which can take various forms, including national health insurance programs, community-based health insurances, and privately-owned health insurance (11, 12). Although health insurance schemes are fraught with diverse challenges among developing countries, research evidence show that they resolve financial barriers to maternal care utilization and enhance access to modern maternal care services (13–15). The positive influence of health insurance on maternal care utilization should be optimally explored to increase the coverage of health facility delivery among women in developing countries (11). Despite the findings by different studies that health insurance schemes can enhance access to maternal care services, however, some of the studies' findings are contradictory, showing that some women who enrolled in health insurance still did not deliver their babies to health institutions (3, 16, 17). This study examines the nature of relationship that exists between health facility delivery and health insurance schemes, necessitating further research on predictors of health facility delivery in Nigeria (11).

**Figure 1:**
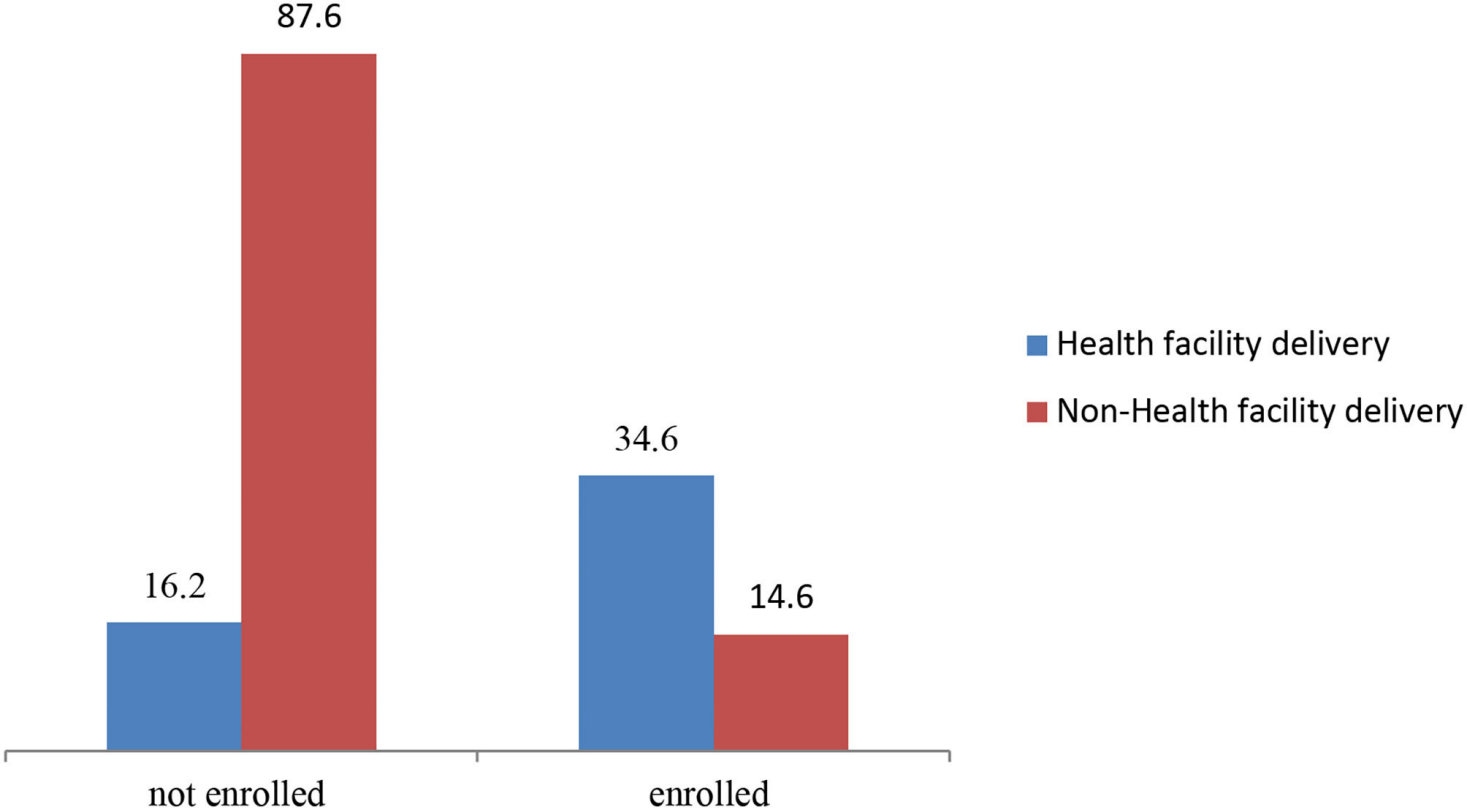
The proportion of women who delivered in health facility across those who enrolled and those who did not enroll in health insurance. Source: Authors'.

Previous studies in Nigeria and other developing countries have reported many individual and household factors that can influence health facility delivery, such as maternal age, maternal education, parity, birth order, attendance at antenatal care (ANC), employment status, sex of heads of households, and pregnancy status (3, 18–23). Studies have also reported a host of community contextual determinants of health facility delivery, such as community poverty level, community media saturation, community literacy level, community fertility norms, community-level urban residence, community-level woman's autonomy, and (24–27). However, it was not certain if this set of factors were uniform determinants of health facility delivery for mothers under health insurance and those not under health insurance. Figure 1 presents the proportion of women who delivered in health facility across those who enrolled and those who did not enroll in health insurance.

A recent Nigerian study (11) revealed different sets of determinants of health facility delivery for mothers under health insurance and those who are not. However, the study did not incorporate community contextual factors as it is a single-level study. This present study extends the scope of the study by incorporating community contextual determinants. The objective of this study is to compare the individual, household, and community-level determinants of health facility delivery for mothers under health insurance and those not under health insurance. The study yields insight on determinants of health facility delivery that are peculiar to women under health insurance and those who are not.

The study is anchored on the health behavioral model by Andersen and Newman (28). The Andersen and Newman behavioral Model (ANBM) for health service utilization provides a framework that permits systematic identification of factors that influence individual decisions to use (or not use) available health care services (28). The authors proposed a theory of healthcare utilization that presented three determinants of healthcare utilization. The determinants are the predisposing, enabling, and need-based characteristics (28, 29). Pre-disposing factors explain the inclination of an individual toward the use of healthcare services before ill-health. They consist of demographic (age, sex, and marital status) variables, social structure (education, occupation, ethnicity, etc.), and health belief. Enabling factors play a supporting role in fulfillment of an individual's need for healthcare. They consist of factors representing the healthcare system characteristics and community resources. Enabling factors (personal and organization) must be present for individuals to utilize healthcare services. Personal enabling factors include income, health insurance, and a regular source of healthcare.

Organizational enabling factors comprise the availability of healthcare providers and their spatial distribution. Need-based characteristics include the perception of needs for health services, whether individual or social or clinically evaluated perception of need (30, 31). Assessment of need can be made by the individual him/herself, by the caregiver, or a health professional based on the symptoms experienced during a period of time and perceived severity of the illness. The analysis in this study draws extensively from the predisposing and enabling factors.

## Materials and Methods

### Data and Sample

The data analyzed in this study was obtained from the National Demographic and Health Survey (NDHS) (32). NDHS (32) was the sixth of its kind to be implemented by its national population commission. The survey was implemented by the National Population Commission with technical and financial support from a number of local and international agencies, such as the National Malaria Elimination Programme (NMEP) of the Federal Ministry of Health (FmOH), Bill and Melinda Gates foundation, and the Global Fund (11). In particular, NDHS (32) used a three-stage sampling stratification, in which respondents were first stratified into rural and urban dwellings. eumeration areas (EAs) were then randomly selected within each stratum. In the third stage, households within each EA were selected using equal probability sampling.

The sampling frame used for the NDHS (32) is the population housing census of the Federal Republic of Nigeria (NPHC) which was used in the 2006 population and National Census. In the NDHS (32), stratification was achieved by separating each of the 36 states and the Federal Capital Territory (FCT) into urban and rural areas. In all, a total of 74 sampling strata were identified. Also, 1,389 EAs were listed and ~30 households were selected in every cluster resulting in a total of 41,821 women being interviewed during the survey, yielding a response rate of 99%. The Demographic and Health Survey (DHS) project was funded by the United States Agency for International Development (USAID) with support from other donors and host countries, and it has conducted over 230 nationally representative and internationally comparable household surveys in more than 80 countries since its inception in 1984 (12, 33).

The data collection procedure and sampling procedure for the survey have been extensively reported elsewhere (33). The data is available and hosted in the public domain (http://dhsprogram.com/data/available-datasets.cfm). This study recruited 6,187 women who reported recent birth in the last 3 years preceding the survey. These women were selected because they have complete responses on the variables of interest and reported recent birth in the last 3 years preceding the survey. The sample size is, therefore, separated into two groups. They are women under health insurance (6,624) and those not under health insurance (137).

### Study Design

This study is a comparative study, which grouped women into two, namely, those enrolled in health insurance and those who did not. This research design helps us to compare the predictors of institutional delivery for mothers under health insurance and those not under health insurance.

### Variables Selection and Measurement

#### Outcome

The dependent and/or outcome variable in the study is health facility delivery (coded 1 for deliveries conducted in health institutions and 0 for deliveries in outside health institutions). Deliveries conducted in public/private hospitals, health centers, and clinics were regarded as health facility delivery (4, 18).

#### Individual Level Factors

Maternal age (15–24/25–34/35–49), maternal education (non-formal/primary education/secondary/higher education), parity (primparity/multiparity/grand multiparity), birth order (1/2/3/4/≥5), pregnancy status (planned/non-planned), employment status (working/non-working), marriage type (monogamy/polygamy), autonomy on healthcare (No/Yes), ownership of land (yes/no), and number of ANC contacts (0/1–3/≥ 4).

#### Household-Level Factors

Sex of household headship (male/female); Household size (1–4/5–7/≥8); Household wealth index was constructed from household assets using principal component analysis (PCA). Households were classified into the following groups: poorest, poorer, average, wealthier, and wealthiest.

#### Community-Level Factors

The study used EAs to represent communities because DHS did not collect aggregate-level data at the community level. Hence, community-level variables included in this study were based on the women's characteristics, particularly those that have implications for the place of delivery. Community-level socioeconomic variable was generated by aggregating the individual-level data to cluster level, except for residential status and geographical region that were taken as they were (34, 35), particularly Residential status (rural /urban) and Geographical region (South South/ South West/ South East/ North Central/ North East/ North West).

Cultural norms about wife-beating within the community surveyed whether half (50%) of the women in the community justified wife-beating for any one of the reasons: if the woman neglects the children, if she argues with the husband, and/or if she denies the husband sex and if burns food. The distribution of uneducated women (illiteracy) within the community was also surveyed. Particularly, whether half (50%) of the women had any form of formal education or not. The concentration of poverty in the community surveyed whether half (50%) of the women within the community falls within the poorest wealth quintiles. The concentration of intimate partner violence within the community surveyed whether half (50%) of the women in the community experienced intimate sexual, psychological, and/or physical partner violence. Lastly, exposure to media surveyed whether half (50%) of the women in the community use any of the print/electronic media (newspaper, magazine, television, radio, or internet). This approach is similar to the methods used by previous studies (34, 35).

### Statistical Analysis

The survey (“svy”) module was used to adjust for stratification, clustering, and sampling weights to compute the estimate of health facility delivery. The characteristics of the women were presented using simple proportions. A multivariable multilevel binary logistic regression was used to estimate the fixed and random effects of the factors associated with health facility delivery. We specified a 3-level model of binary responses reporting place of delivery, namely, level 1 (individual-level factors), level 2 (household level factors), and level 3 (community-level factors). We estimated also five models. The first model is an empty or null model without explanatory variables (random intercept), the second model controlled only for individual-level factors, the third model controlled for household-level factors, the fourth model controlled for community-level factors, and model five simultaneously controlled for individual, household, and community-level factors. For all models, the study presented the adjusted odds ratio and associated 95% confidence intervals. Separate analyses were undertaken for women under health insurance and those not under health insurance. These models were fit with Stata command for identification of variables that were statistically significant in explaining health facility delivery. For model comparison, we utilized optimality criteria which include Akaike Information Criterion (AIC) and log-likelihood ratio. A model with the smallest AIC was adjudged to have the best fit (35, 36). The manuscript was written by following the Strengthening Reporting of Observational Studies in Epidemiology (STROBE) reporting guidelines (37).

### Ethical Consideration

The study used secondary data hoisted on a public domain with all identifier information removed. The authors were granted access to the data set by MEASURE DHS/Informed consent form (ICF) international. ICF international ensures that the survey complies with the U.S. Department of Health and Human Services regulations for the respect of human subjects. No ethical clearance was retrieved for the study. More details about data and ethical standards are available at http://goo.gl/ny8T6X.

## Results

### Respondents' Socio-Demographic Characteristics

Table 1 presents the sociodemographic characteristics of the respondents. A higher proportion of respondents are within the age brackets of 35–39 years regardless of health insurance enrollment. While majority of mothers who were not under health insurance reported non-formal and primary educational qualifications, majority of them who were under health insurance reported secondary and tertiary educational qualifications. For both groups, a higher proportion of the women were employed and working. Majority of the women did not own land regardless of health insurance enrollment. Multi-parous mothers were dominant among the respondents. However, the proportion of grand multiparous mothers was higher among women who do not enroll in health insurance compared to those who enrolled.

**Table 1:** Sociodemographic characteristics of respondents.

**Variables**	**Women not under health insurance** **(*n* = 137)**	**Women under Health Insurance** ** (*n* = 6,624)**
	**Percentage (%)**	**Percentage (%)**
**Maternal age (years):**		
15–24	25.7	20.1
25–34	35.2	39.6
35–49	39.1	40.3
**Maternal education:**		
Non-formal	10.1	9.6
Primary	20.3	12.9
Secondary	31.2	32.4
Higher	38.4	45.1
**Employment status:**		
Not working	40	46
Working	60	54
**Ownership of land:**		
No	53	67.7
Yes	47	32.3
**Birth order:**		
1	20.8	25.6
2	25.6	24.5
3	26	23.7
4	38.4	15
≥5	19.4	11.2
**Marriage type:**		
Monogamy	53.6	66
Polygamy	46.4	34
**Parity:**		
Prim parity (1)	20.2	20.4
Multiparity (2–4)	45.4	40.4
Grand multiparity (≥5)	43	39
**Autonomy on healthcare:**		
No	47	47.3
Yes	53	52.7
**Pregnancy status:**		
Planned	49	52.3
Non-planned	51	47.7
**Number of ANC contacts:**		
No visits	12.7	15
1–3 visits	28.8	25
≥4	58.5	69
**Household characteristics:**		
**Sex of household head:**		
Male	40	45.2
Female	60	54.8
**Household wealth quintile:**		
Poorest	30.4	3.1
Poorer	27.8	12.8
Average	20.3	25.5
Wealthy	15.7	25.8
Wealthiest	5.8	32.8
**Household size:**		
4-Jan	35	24.2
≥5	65	75.8
**Residential status:**		
Urban	40.2	62.9
Rural	59.8	37.1
**Geopolitical zones:**		
North	20.8	43.2
South	79.2	56.8
**Cultural norm for wife beating:**		
No	56.2	65.2
Yes	43.8	34.8
**Community-level poverty:**		
Low	30.9	20
High	69.1	80
**Community-level media use:**		
Low	56.8	13.8
High	43.2	65.9
**Community level literacy:**		
Low	68.1	24.1
High	41.9	75.8
**Community—level of woman's autonomy**		
Low	66.8	33.6
High	33.2	66.4
**Health facility delivery:**		
No	67.6	35.7
Yes	32.4	64.3

*Source: Authors'*.

For both groups of women, majority of them were in monogamous marriages. A higher proportion of the respondents reported a birth order of two across both groups. Regardless of health insurance enrollment, majority of the women reported that they planned their pregnancies. Across both groups, majority of the women reported autonomy in their own healthcare decisions. Distribution by household wealth quintile shows that women who had health insurance were better off with the majority of them located in the wealthiest quintiles compared to the non-enrolled group in which the majority of them were in the poorest wealth quintile. For both groups, majority of the mothers were drawn from male-headed households.

Higher proportions of the respondents reported ≥4 antenatal contacts in the two groups. However, the proportion seems higher among women who enrolled in health insurance than those not enrolled. While majority of respondents who are not under health insurance resides in rural areas, majority of those who enrolled reside in the urban parts of the country. Regardless of insurance ownership, majority of the women were drawn from northern parts of the country. Majority of respondents who were not enrolled in a health insurance scheme was drawn from communities with cultural norms for wife beating, high community poverty level, low community media use, low community literacy rate, and low women's autonomy level. On the contrary, a high proportion of respondents who enrolled in health insurance scheme were drawn from communities without cultural norms for wife-beating, low community poverty level, high community media use, high community literacy rate, and high women's autonomy level.

### Determinants of Health Facility Delivery Among Women Not Enrolled in Health Insurance

Table 2 shows the fixed and random effects result on predictors of health facility delivery for women not enrolled in health insurance among childbearing women in Nigeria. The results from the fixed effect models showed that significant predictors of health facility delivery were maternal education, employment status, marriage type, autonomy on healthcare, number of ANC contacts, household wealth quintiles, residential status, geopolitical zones, community-level poverty, and community-level media use. Respondents who reported primary educational qualifications (AOR = 2.6, 95% CI: 0–0.1), secondary educational (AOR = 2.9; 95% CI:0.1–2.1) qualifications, and higher educational qualifications (AOR = 3.8, 95% CI: 0–3.4) were significantly more likely to deliver their babies in health facilities compared to those who had non-formal education. In reference to mothers not working, those of them employed (AOR = 3.8, 95% CI: 0–1.4) were approximately four times as likely to deliver in health institutions.

**Table 2:** Fixed effects of individual, household, and community-level factors associated with health facility delivery among women not enrolled in health insurance.

**Variables**	**Model I**	**Model II**	**Model III**	**Model IV**	**Model V**
**Maternal age (years):**					
15–24 25–34 35–49		1.0 2.7 (0.1–0.9) 3.8 (0.8–1.2)			1.0 2.8 (0.0–0.8) 3.8 (0.0–1.2)
**Maternal education:**					
Non-formal Primary Secondary Higher		1.0 1.8 (0.0–1.1)^*^ 2.2 (0.1–1.7)^*^ 3.4 (0.2–1.9)^*^			1.0 2.6 (0.0–0.1)^*^ 2.9 (0.1–2.1)^*^ 3.8 (0.0–3.4)^*^
**Employment Status:**					
Non-working Working		1.0 3.7 (0.0–1.8)^*^			1.0 3.8 (0.0–1.4)^*^
**Ownership of land:**					
No Yes		1.0 3.8 (1.1–2.3)			1.0 7.8 (0.0–1.1)
**Birth order:**					
1 2 3 4 ≥5		1.0 1.1 (0.0–1.1) 2.3 (0.2–1.7) 3.7 (0.0–0.0) 4.8 (1.1–2.3)			1.0 1.1 (0.0–1.2) 2.3 (0.1–3.4) 3.6 (0.1–2.5) 3.8 (1.1–3.4)
**Marriage type:**					
Monogamy Polygamy		1.0 2.8 (0.0–1.8)^*^			1.0 0.1(0.0–1.1)^*^
**Parity:**					
Prim parity (1) Multiparity (2–4) Grand Multiparity (≥5)		1.0 2.4 (0.5–9.8) 2.8 (0.0–1.8)			1.0 2.3 (0.0–1.1) 2.6 (0.0–1.8)
**Autonomy on healthcare:**					
No Yes		1.0 3.4 (0.0–1.8)^*^			1.0 2.6 (0.1–1.8)^*^
**Pregnancy status:**					
Planned Non-planned		1.0 2.2 (0.1–4.3)^*^			1.0 2.3 (0.9–1.8)
**Number of ANC contacts:**					
No visits 1–3 visits ≥4		1.0 1.1 (0.8–1.2) 2.8 (0.0–0.8)^*^			1.0 1.8 (0.9–1.8)^*^ 2.8 (0.0–1.8)^*^
**Sex of household head:**					
Male Female		1.0 2.9 (0.0–1.1)			1.0 2.3 (0.0–1.1)
**Household wealth quintile:**					
Poorest Poorer Average Wealthy Wealthiest			1.0 1.8 (0.0–1.2) 2.1 (0.0–1.9) 3.8 (0.0–0.0) 4.8 (0.0–1.2)		1.0 2.1 (0.0–1.2)^*^ 3.4 (0.0–0.0)^*^ 3.8 (0.0–1.1)^*^ 4.8 (0.1–1.6)^*^
**Household size:**					
1–4 ≥5			1.0 1.9 (0.0–1.3)^*^		1.0 1.1 (0.2–2.3)
**Residential status:**					
Rural Urban				1.0 2.8 (0.0–0.0)^*^	1.0 2.6 (0.0–1.0)^*^
**Geopolitical zones:**					
North South				1.0 2.3 (0.0–1.1)^*^	1.0 4.8 (0.0–1.2)^*^
**Cultural norm for wife beating:**					
No Yes				1.0 1.8 (0.0–1.1)	1.0 1.8 (0.0–1.0)
**Community-level poverty:**					
Low High				1.0 0.23 (0.0–0.1)^*^	1.0 0.3 (0.0–1.6)^*^
**Community-level media use:**					
Low High				1.0 2.1 (0.0–0.8)^*^	1.0 2.9 (0.0–1.1)^*^
**Community level literacy:**					
Low High				1.0 3.4 (0.0–0.8)	1 0.4 (0.9–0.8)
**Community -level of woman's autonomy**					
Low High				1.0 1.8 (0.0–1.2)	1.0 2.4 (0.0–1.8)
**Random effects:**					
PSU Variance (95% CI)	2.0 (0.7–5.1)	2.4 (1.0–5.2)	2.1 (0.9–0.56)	3.1 (0.8–4.7)	4.1 (0.1–2.7)
ICC	0.52	0.39	0.40	0.39	0.42
LR Test	X^2^ = 1,395.0 *P* < 0.001^*^	X^2^ = 609.42 *P* < 0.001^*^	X^2^ = 377.75 *P* < 0.001^*^	X^2^ = 299.8 *P* < 0.001^*^	X^2^ = 477.48 *P* < 0.001^*^
Wald X^2^	**References**	309.67^*^	234.56^*^	543.78^*^	321.78^*^
**Model fitness:**					
Log-likelihood	−2,900.4	−2,748.8	−2,491.4	−2,841.6	−3,194.8
AIC	3,418.3	3,142.7	2,179.8	2,134.0	1,989.4
Sample size	6,624	6,624	6,624	6,624	6,624

*Source: 2018 Nigeria Demographic and Health Survey*.

*Model 1: is the null model, a baseline model without any determinant variable; Model 2: is adjusted for individual-level variables; Model 3: is adjusted for household variables; Model 4: is adjusted for community-level variables; Model 5: adjusted for all four variables (individual, household and community-level); aOR, Adjusted odds ratios; CI, Confidence interval; Ref, Reference category; PSU, Primary Sampling Unit; ICC, Intra-Class Correlation; LR Test, Likelihood ratio Test; AIC, Akaike's Information Criterion*.

* *Significant at 5%*.

Compared to mothers in monogamous types of marriage, those in polygamous marriage (AOR = 0.1, 95% CI:0.1–1.1) were 90% significantly more likely to deliver in health institutions. Mothers who reported 1–3 ANC contacts (AOR = 1.8, 95% CI:0.9–1.8) and ≥4 ANC contacts (AOR = 2.8, 95% CI: 0–1.8) were more likely to deliver in health institutions compared to those who made no contacts. The odds for health facility delivery improves all throughout household wealth quintile. Hence, those located in poorer wealth quintile (AOR = 2.1, 95% CI: 0–1.2), average wealth quintile (AOR = 3.4, 95% CI: 0), wealthy quintile (AOR = 3.8, 95% CI: 0–1.1), and wealthiest quintile (AOR = 4.8, 95% CI:0.1–1.8) were significantly more likely to deliver in health facilities compared to those located in the poorest wealth quintile. Urban respondents (AOR = 2.6, 95% CI: 0–1.0) were more likely to deliver in health institutions when compared to mothers drawn from rural parts of the country. Mothers drawn from Southern Nigeria (AOR: 2.6, 95% CI: 0–1.2) were more likely to deliver in health institutions compared to mothers from northern parts of the country.

In reference to mothers drawn from communities with low media use, those drawn from communities with high media use (AOR: 2.9, 95% CI; 0–1.1). In reference to respondents drawn from community with low poverty ranking, those from communities with high poverty rankings (AOR: 0.3; 95% CI: 0–1.6) were 70% significantly less likely to deliver in health institutions. With the random effects result, the full model (Model 5) which controls for individual characteristics, household characteristics, and community-level factors has the lowest Akaike Information Criterion (1,989.4) with a log-likelihood ratio of −3,194.8. Hence, it was considered to be the line of best fit suitable for predicting health facility delivery among the women.

### Determinants of Health Facility Delivery Among Women Enrolled in Health Insurance

Table 3 shows the fixed and random effects result on predictors of health facility delivery for women enrolled in health insurance among childbearing women in Nigeria. In terms of the fixed effect results, we found out that in reference to mothers without formal education, those with primary educational qualifications (AOR = 1.2, 95% CI: 0–0.8), secondary educational qualifications (AOR: 1.8, 95% CI: 0–1.2), and higher educational qualifications (AOR: 2.2, 95% CI:0.4–4.5) were significantly more likely to deliver their babies in health institutions. Also, in reference to mothers who reported a birth order of 1, those who reported 4 children (AOR: 0.8, 95% CI: 0–1.1) were less likely to deliver their babies in health institutions, while those who reported ≥ 5 (AOR = 4.3, 95% CI: 0–1.2) were more likely to deliver their babies in health institutions. In reference to mothers who reported primiparity (1 child), those who were multiparous (AOR: 0.8, 95% CI: 0–1.1) and grand-multiparous (AOR: 0.9, 95% CI: 0) were less likely to deliver their babies in health institutions. In reference to mothers who reported no ANC contacts, those who reported 1–3 (AOR: 2.9; 95% CI: 0–1.0) and ≥4 (AOR: 3.4; 95% CI:0.6–1.4) were significantly more likely to deliver their babies in health institutions. In reference to those who belonged to the poorest wealth quintile, those who belonged to the poorer (AOR: 1.2, 95% CI: 0–1.2), average (AOR: 1.8; 95% CI: 0), wealthier (AOR1.9, 95% CI.1–2.3), and wealthiest (AOR: 2.8, 95% CI: 0–1.2) were significantly more likely to deliver their babies in health institutions. In reference to respondents drawn from community with low poverty ranking, those of them from communities with high poverty ranking (AOR: 0.8; 95% CI: 0–1.1) were 20% significantly less likely to deliver in health institutions. In reference to mothers drawn from communities with low media use, those drawn from communities with high media use (AOR: 2.2, 95% CI: 0–2.5) were significantly more likely to deliver their babies in health institutions. Finally, mothers who resided in communities with high literacy ratings (AOR: 2.7; 95% CI: 0–0.9) were more likely to deliver their babies in health institutions compared to mothers from communities with low literacy ratings. With the random effects result, the full model (Model 5) which controls for individual characteristics, household characteristics, and community-level factors has the lowest Akaike Information Criterion (1,089.4) with a log-likelihood ratio of −3,184.9. Hence, it is considered the line of best fit and considered most suitable for predicting health facility delivery among the women.

**Table 3:** Fixed effects of individual, household, and community level factors associated with health facility delivery among women enrolled in health insurance.

**Variables**	**Model I**	**Model II**	**Model III**	**Model IV**	**Model V**
**Maternal age (years):**					
15–24 25–34 35–49		1.0 2.3 (0.8–6.7) 3.6 (0.0–1.0)			1.0 1.8 (0.0–1.0) 2.2 (0.5–9.8)
**Maternal education:**					
Non-formal Primary Secondary Higher		1.0 1.8 (0.0–1.8)* 1.9 (0.0–2.0)* 2.3 (0.0–1.0)*			1.0 1.2 (0.0–0.8)* 1.8 (0.0–1.2)* 2.2 (0.4–4.5)*
**Employment status:**					
Non-working Working		1.0 2.5 (0.0–1.1)			1.0 2.3 (0.0–2.3)
**Ownership of land:**					
No Yes		1.0 1.9 (0.0–1.8)			1.0 1.1 (0.0–1.0)
**Birth order:**					
1 2 3 4 ≥5		1.0 2.3 (0.0–0.2) 2.6 (0.0–2.3) 2.8 (0.0–0.0)* 3.1 (0.0–1.2)*			1.0 2.3 (0.0–1.2) 2.8 (0.0–0.1) 3.4 (0.0–1.2)* 4.3 (0.0–1.2)*
**Marriage Type:**					
Monogamy Polygamy		1.0 2.3 (0.9–3.8)			1.0 3.4 (0.0–2.2)
**Parity:**					
Prim parity (1) Multiparity (2–4) Grand Multiparity (≥5)		1.0 0.8 (0.0–1.1)* 0.9 (0.0–0.0)*			1.0 2.3 (0.0–1.1)* 2.8 (0.0–0.0)*
**Autonomy on healthcare:**					
No Yes		1.0 1.8 (0.0–1.1)			1.0 3.8 (0.0–1.2)*
**Pregnancy status:**					
Planned Non-planned		1.0 0.8 (0.0–1.0)*			1.0 2.3 (0.0–9.8)
**Number of ANC contacts:**					
No visits 1–3 visits ≥4		1.0 (0.0–1.2)* 1.3 (0.0–0.9)* 2.4 (0.0–0.0)*			1.0 2.9 (0.0–1.0)* 3.4 (0.0–1.4)*
**Sex of household head:**					
Male Female			1.0 2.3 (0.0–0.8)		1.0 3.4 (0.0–1.2)
**Household wealth quintile:**					
Poorest Poorer Average Wealthy Wealthiest			1.0 1.8 (0.8–7.8)* 2.6 (0.1–0.1)* 3.7 (0.0–0.7)* 4.3 (0.0–0.0)*		1.0 1.2 (0.0–1.2)* 1.8 (0.0–0.0)* 1.9 (0.1–2.3)* 2.8 (0.0–1.2)*
**Household size:**					
1–4 ≥ 5			1.0 2.9 (0.0–0.9)		1.0 1.2 (0.0–1.2)
**Residential status:**					
Rural Urban				1.0 2.8 (0.0–9.8)*	1.0 2.9 (0.0–1.2)*
**Geopolitical zones:**					
North South				1.0 3.8 (0.0–1.5)	1.0 1.8 (0.0–0.9)
**Cultural norm for wife beating:**					
No Yes				1.0 2.1 (0.0–0.9)	1.0 3.4 (0.1–2.3)
**Community–level poverty:**					
Low High				1.0 4.8 (0.0–3.4)*	1.0 0.8 (0.0–1.1)*
**Community-level media use:**					
Low High				1.0 5.6 (0.0–0.0)*	1.0 2.2 (0.0–2.5)*
**Community level literacy:**					
Low High				1.0 2.6 (0.0–0.8)*	1.0 2.7 (0.0–0.9)*
**Community–level of woman's autonomy**					
Low High				1.0 2.6 (0.9–1.8)	1.0 3.6 (0.0–2.3)
**Random effects:**					
PSU Variance (95% CI)	3.0 (0.8–5.2)	1.4 (1.0–3.2)	3.1 (0.9–0.66)	3.5 (0.8–6.7)	4.8 (0.1–3.7)
ICC	0.52	0.49	0.50	0.49	0.52
LR Test	X^2^ = 1,115.0 *P* < 0.001*	X^2^ = 509.42 *P* < 0.001*	X^2^= 477.75 *P* < 0.001*	X^2^ = 199.8 *P* < 0.001*	X^2^ = 277.48 *P* < 0.001*
Wald X^2^	**References**	409.57*	334.56*	443.88*	421.88*
**Model fitness:**					
Log-likelihood	−3,900.8	−3,848.8	−3,481.4	−3,881.6	−3,184.9
AIC	3,318.3	2,142.7	2,479.8	1,134.0	1,089.4
Sample size	137	137	137	137	137

*Source: 2018 Nigeria Demographic and Health Survey*.

Model 1: is the null model, a baseline model without any determinant variable; Model 2: is adjusted for individual-level variables; Model 3: is adjusted for household variables; Model 4: is adjusted for community-level variables; Model 5: adjusted for all four variables (individual, household and community-level); aOR, Adjusted odds ratios; CI, Confidence interval; Ref, Reference category; PSU, Primary Sampling Unit; ICC, Intra-Class Correlation; LR Test, Likelihood ratio Test; AIC, Akaike's Information Criterion. *Significant at 5%.

## Discussion of the Results

In this study, we compared determinants of health facility delivery for mothers under health insurance schemes and those not under the health insurance scheme. Secondary data from the most recent NDHS (32) was used for the analysis. The data is hoisted on a public domain, hence making the research results reproducible. The study is anchored on the Andersen and Newman (28) behavioral model. The findings of the study validated the proposition made by this model and other studies that utilized the model.

The result showed that the prevalence of health insurance enrollment among women is low. For instance, only 2.1% (137/6,761) of mothers were under health insurance scheme. The result is in conformity with results from other studies that reported low prevalence of health insurance among reproductive age women among developing countries (4, 9, 11–13, 38). For Nigeria to benefit optimally from the implementation of health insurance schemes, health planners and authorities must device other means of encouraging widespread enrollments in health insurance schemes, particularly among poorer and rural women (11). Given the restricted coverage of the Nigeria National health insurance program, the Nigerian government should consider reprogramming the national health insurance program so that it can provide coverage for women in the informal sector (13). The government should make efforts to encourage improvement in community-based health insurance schemes in Nigeria, with the intent to encourage trust among enrollees, effective delivery of services, and affordability of premium (13).

We noted that the rate of health facility delivery is low among the respondents. This result is in conformity with results from past studies both for Nigeria and elsewhere (11, 32, 39, 40). This suggests that eliminating the practice of non-institutional delivery is a major public health challenge in Nigeria (32). In Nigeria, there is a high rate of home deliveries, particularly in rural and remote parts of the country (11, 41, 42). It is therefore imperative that intervention program design to improve the coverage of health facilities should be implemented. Health education and awareness program should be used to encourage mothers to deliver in health institutions.

The result showed that there is a difference in the coverage of health facility delivery between women who enrolled into health insurance schemes and those who do not. According to the data, the coverage of health facility delivery for mothers enrolled in health insurance is more than twice those who did not enroll in health insurance. There are also slight differences in the determinants of health facility delivery for both groups. For instance, unique predictors of health facility delivery for enrolled women were birth order and parity, while unique determinants of health facility delivery for the non-enrolled group were employment status, marriage type, and geopolitical zones. This differences in the enrolled group and non-enrolled group are due to differences in their socioeconomic conditions (11). As noted in this study, disparity exists in health insurance ownership by Nigerian women, with the better educated, those in urban areas, and those from wealthier and wealthiest quintile better off in health insurance ownership.

This report is in tandem with reports made by past studies both for Nigeria and other countries (11, 13, 43). Fenny, Yates, and Thomson, (44) in a study for a set of African countries, revealed marginalization of the poor from health insurance services. We recommended that large-scale research should be conducted in Nigeria that will explore how the poor and uneducated women can benefit from health insurance programs. Policymakers in sub-saharan Africa (SSA) should pay more attention to the marginalization and vulnerable sectors of the society, and explore the potency of health insurance programs in increasing maternal healthcare utilization (43). We noted that household wealth quintiles, maternal education, place of residence, number of ANC contacts, community-level poverty, community-level media use, and community-level literacy were uniform determinants of health facility for both women enrolled in health insurance and those who do not enroll.

Household wealth has been reported as a significant determinant of health facility delivery by past studies for Nigeria and elsewhere (20, 45, 46). The reason for this relationship is that health facility delivery involves a lot of costs that women from improved economic households can more easily pay for (35). Even when the government implements free delivery cares, there are other direct and indirect charges that will make women from the high socioeconomic background better off. This result suggests the use of multi-pronged approach to improve household socioeconomic conditions in Nigeria (45).

We noted that maternal education has a significant positive impact on institutional delivery for both enrolled women and non-enrolled women. This result is in conformity with those of other studies conducted in Nigeria and elsewhere (19, 21, 22, 45). There are many pathways through which education may impact on women's decision to use health facilities in times of delivery. These pathways include empowerment and confidence to negotiate with their husband and healthcare providers, higher employment opportunities, higher chances to live in urban areas close to health facilities, and knowledge associated with the use of modern healthcare services (35, 46). Also, maternal education can improve healthcare literacy. Thus, they are more aware of the danger signs and may be able to identify signs associated with labor (33). This result suggests that the Nigerian government should expand education opportunities for mothers within the reproductive ages. From the result, a minimum of primary education should be the baseline.

We noted that women residing in urban parts of Nigeria were more likely to deliver their babies in health institutions compared to those in rural parts for both the enrolled group and the not-enrolled group. This same finding was reported by past studies (22, 47). The result suggests that urban women have an advantage over their rural counterparts, influencing them to positively use maternal care services. Differences in socioeconomic development, educational attainments, and accessibility to health facilities may be responsible for the observed geographical variation (38, 45, 48). Another reason may be inequalities in the distribution of accessible health resources between rural and urban parts of the country (33). In rural parts of Nigeria, there are fewer health facilities, and may not be accessible due to poor road network, inefficient transport, and long-distance (49). Also, health systems in rural areas are not adequately financed, hence it may not be able to attract and retain competent health workers (40). Furthermore, women residing in rural parts of the country may be more influenced by cultural beliefs and social norms that may discourage delivery in health facilities (49).

It is therefore imperative that efforts to increase health facility delivery among Nigerian women should focus more on rural women. We found out that women who reported ≥4 ANCs are more likely to use health facilities as a place of delivery for both the enrolled group and non-enrolled group. The result is in harmony with results of past studies (13, 17, 49). This is because adequate attendance at ANC avails women the opportunity to learn the benefits of health facility delivery and encourage them to do so (11, 50). At the community level, community media-use, community literacy level, and community poverty level were the significant predictors of health facility delivery for both mothers enrolled in health insurance and those who did not enrolled. These results are in harmony with the results reported by and confirmed the role of community contextual determinants on health facility delivery among Nigerian women.

## Limitations of the Study

This study is not without limitations. Some of the limitations are as follows: (i) the relatively small number of women that enrolled in health insurance did not allow for more rigorous comparison. For instance, the study could not explore the potential effects of health insurance on health facility delivery for this reason. However, the difference in the number of those who enrolled and those who did not may not have affected the results because separate analyses were undertaken for both groups of women; (ii) given the cross-sectional nature of the data, the study could not establish a cause-effect relationship as only association was established; (iii) the data belonged to different dates and times. For instance, responses on health facility delivery were limited to 5 years prior to the survey, but information on sociodemographic factors were based on the time respondents were interviewed; (iv) the study engaged the logit regression analysis, and as a result, could not control for endogeneity. Therefore, future studies should include community contextual factors as determinants of facility delivery for both groups of women. Despite these limitations, the study has yielded useful insight into predictors of health facility delivery among women health insurance enrollment and non-enrollment.

## Conclusion

Drawing upon secondary data from the most recent National Demographic and Health Survey, (32) we have showed that the prevalence of health insurance enrollment among reproductive-age women is abysmally low. The data also showed disparity in health insurance ownership with mothers drawn from wealthy homes, more educated mothers, and those residing in urban parts of the country to be better off in health insurance enrollment. Furthermore, a high proportion of mothers under health insurance schemes delivered in health institutions compared to those not under the scheme.

The logistic regression results showed that unique determinants of health facility delivery for mothers enrolled in health insurance schemes were in parity and birth order, while for those not under health insurance were employment status, marriage type, and geopolitical zones. Also, uniform determinants of health facility delivery were maternal education, household wealth quintiles, a minimum of four antenatal contacts, and place of residence. This suggests that intervention program design to improve coverage of health facility delivery in Nigeria should expand education opportunities for mothers, improve household socioeconomic conditions by implementing pro-poor program, target poor rural women, and encourage mothers to undertake a minimum of four antenatal contacts among pregnant mothers.

## Data Availability Statement

Publicly available datasets were analyzed in this study. The data is available on the DHS site.

## Author Contributions

RA designed the study. All authors reviewed the literature and approved the final draft.

## Conflict of Interest

The authors declare that the research was conducted in the absence of any commercial or financial relationships that could be construed as a potential conflict of interest.

## Publisher's Note

All claims expressed in this article are solely those of the authors and do not necessarily represent those of their affiliated organizations, or those of the publisher, the editors and the reviewers. Any product that may be evaluated in this article, or claim that may be made by its manufacturer, is not guaranteed or endorsed by the publisher.
